# Healthcare Worker Preferences for Active Tuberculosis Case Finding Programs in South Africa: A Best-Worst Scaling Choice Experiment

**DOI:** 10.1371/journal.pone.0133304

**Published:** 2015-07-21

**Authors:** Nathan N. O’Hara, Lilla Roy, Lyndsay M. O’Hara, Jerry M. Spiegel, Larry D. Lynd, J. Mark FitzGerald, Annalee Yassi, Letshego E. Nophale, Carlo A. Marra

**Affiliations:** 1 School of Population and Public Health, University of British Columbia, Vancouver, British Columbia, Canada; 2 Collaboration for Outcomes Research and Evaluation, Faculty of Pharmaceutical Sciences, University of British Columbia, Vancouver, British Columbia, Canada; 3 Institute for Heart and Lung Health, University of British Columbia, Vancouver, British Columbia, Canada; 4 Department of Community Health, Faculty Of Health Sciences, University of the Free State, Bloemfontein, South Africa; 5 School of Pharmacy, Memorial University, St. John’s, Newfoundland, Canada; Johns Hopkins Bloomberg School of Public Health, UNITED STATES

## Abstract

**Objective:**

Healthcare workers (HCWs) in South Africa are at a high risk of developing active tuberculosis (TB) due to their occupational exposures. This study aimed to systematically quantify and compare the preferred attributes of an active TB case finding program for HCWs in South Africa.

**Methods:**

A Best–Worst Scaling choice experiment estimated HCW’s preferences using a random-effects conditional logit model. Latent class analysis (LCA) was used to explore heterogeneity in preferences.

**Results:**

“No cost”, “the assurance of confidentiality”, “no wait” and testing at the occupational health unit at one’s hospital were the most preferred attributes. LCA identified a four class model with consistent differences in preference strength. Sex, occupation, and the time since a previous TB test were statistically significant predictors of class membership.

**Conclusions:**

The findings support the strengthening of occupational health units in South Africa to offer free and confidential active TB case finding programs for HCWs with minimal wait times. There is considerable variation in active TB case finding preferences amongst HCWs of different gender, occupation, and testing history. Attention to heterogeneity in preferences should optimize screening utilization of target HCW populations.

## Introduction

Occupational exposure to tuberculosis (TB), including multiple and extensively-drug resistant TB (MDR-TB and XDR-TB) poses a serious risk to all healthcare workers (HCWs) globally.[1–3] South Africa has the second highest incidence of TB in the world at 1000 per 100,000, a rate that has increased more than five-fold since 1986.[4–6] The emergence of MDR-TB and XDR-TB has heightened the importance of improving access to and utilization of active TB case finding programs, as well as the provision of effective treatment to prevent the spread of this highly infectious disease.[7] HCWs in South Africa face a very high risk of acquiring TB in the workplace due to high rates of TB, MDR-TB and XDR-TB exposure combined with a high prevalence of human immunodeficiency virus (HIV) among HCWs and deficiencies in infection control practices.[6,8–10] Studies suggest that despite the fact that HCWs are at high-risk of occupational exposure to blood-borne and airborne infections, the timeliness of diagnosis and prompt initiation of treatment among this group remain low.[11–16]

To ensure the health and safety of HCWs internationally, the American College of Occupational and Environmental Medicine (ACOEM) recommends that all health facilities incorporate TB surveillance for HCWs using baseline and periodic screening.[17] Due to the high prevalence of latent TB infection (LTBI) in South Africa, case finding and TB testing policies focus on active TB disease as opposed to LTBI.[4,5] Most health facilities in South Africa do have occupational health units (OHUs) available to provide free testing for active TB, however, it is thought that healthcare workers are often not tested regularly and one study found that only 32% of HCWs in Free State South Africa had ever been tested for active TB.[15,18] Even HCWs without routine patient contact can be at an elevated risk for TB exposure. A recent study that collected air samples in a South African hospital noted concerning levels of *Mycobacterium tuberculosis* in areas not used for patient care, such at the information technology room.[19]

The ultimate goal of an active TB case finding program is to promote early and accurate TB diagnosis, which is essential to improve treatment outcomes for individual patients and to reduce transmission to others.[5,20] In 2010, the WHO, International Labour Organization, and Joint United Nations Program on HIV/AIDS released guidelines recommending that testing for active TB be available to HCWs and combined with other infection-control interventions.[21] The systematic review conducted during the WHO guideline development process highlighted the fact that there are few published studies focused specifically on issues related to testing programs for this high-risk workforce.

Although the South African National Department of Health has comprehensive TB guidelines that address active TB case finding,[22,23] HCW case finding programs are designed and implemented provincially. Programs also vary considerably by hospital, and in many cases, are incomplete or not implemented at the hospital level. Frequency and design of HCW case finding programs in each hospital depends on numerous items, such as organizational culture, managerial support and availability of occupational health staff.

Active TB case finding programs must be organized in congruence with HCWs preferences to optimize participation. As such, the objective of our study was to elicit the preferences of HCWs in South Africa pertaining to active TB case finding.

## Methods

### Study design

We employed a survey consisting of choice experiment questions in a sample of HCWs in the Free State Province of South Africa in January 2013. Choice experiments work on the premise that any ‘product’, for example a healthcare treatment or drug therapy, can be described by levels of its characteristics, known as attributes. The extent to which an individual values the ‘product’ is dependent on a weighted sum of the levels of these characteristics.[24] Choice experiments are underpinned by random utility theory, [25] which states that the probability that product A is chosen over product B is proportional to how much product A is valued over product B. Choice experiments were developed in marketing research but have become increasingly popular in health services research and have been used to explore a range of health related services and treatments.[26–28] There are two types of choice experiments typically utilized–Discrete Choice Experiments (DCE) and Best-Worst Scaling (BWS). In a BWS choice experiment, individuals choose the best and the worst attribute based on the levels displayed in a given specification and preferences can be ascertained from their responses.[29,30] Because two opposing choices (best and worst) are selected for each question, research has shown comprehension, reliability of response, and design efficiency is improved.[31] A BWS choice experiment was selected for this study over a DCE for these reasons, as well as the fact that the analysis allows for the utility of each attribute level to be compared to a single, common reference level, providing additional insight for knowledge translation to policy makers.

### Ethics statement

Ethics approval for this study was obtained at the University of British Columbia (UBC) and from the Ethics Committee of the Faculty of Health Sciences at the University of the Free State (UFS). Informed written consent was obtained from all participants in accordance with UBC and UFS consenting procedures. The study was conducted in the context of a collaborative Canadian—South African global health research program to improve the health of health workers, building upon established relationships and commitments for on-going partnership.[32,33]

### Attribute development

To derive the attributes for use in the BWS, qualitative methods are recommended.[29]

The attributes included in this experiment were identified through key informant interviews of HCWs (n = 3) and three focus groups (n = 40). Two focus groups comprised of HCWs (n = 15) of various professions currently employed in two public hospitals in the Free State Province of South Africa. The third focus group comprised of physicians (n = 25) who had a faculty affiliation with the University of the Free State. HCWs were selected for the focus groups based on a convenience sample. All focus groups were audio recorded, transcribed verbatim, and coded for attributes and attribute levels. Each attribute is comprised of levels that become a potential factor in the BWS choice set. Attributes and levels were reduced to the minimum number deemed important by qualitative data, expert opinion, and clinical relevance. Table 1 lists the attributes that emerged from the focus groups as the most important factors in a HCWs’ decision for TB testing. Although TB testing is currently available without a fee to all HCWs through the public health system, it was noted in the focus groups that many HCWs still seek TB testing through their private general practitioner where they may incur a fee. Cost was therefore included as an attribute in the questionnaire.

**Table 1:** Attributes and levels included in the best worst scaling experiment.

Attribute	Attribute levels
Test location	At your place of employment
	At a clinic in the community where you live
	At a clinic in another community where you live
Wait time	No wait
	2 hours
	4 hours
	6 hours
Cost^1^	No cost
	R100
	R200
	R300
Tested by	Physician
	Registered nurse
	Allied health worker
Confidentiality^2^	Yes
	No

^1^ $1 CDN = R 9.02, as per January 31, 2013

^2^ Confidentiality implies that the participation of the healthcare worker in a TB test will remain private and not be disclosed to the hospital or another third party.

### Experimental design and construction of choice sets

The attributes selected were used to design a BWS questionnaire in Sawtooth software (Sawtooth Software, Inc. Sequim, WA, USA). Including five attributes and a total of 16 attribute levels gave a full factorial of 288 possible combinations. To provide a manageable task for respondents, we used the D-optimality criterion to maximize the efficiency of the design. The final design was based on four versions of the survey. Each survey consisted of 12 choice sets.

### Best-Worst Scaling survey

There are no formal sample size calculations for choice experiments, but a conventional heuristic recommends 50 participants per sub-group in the analysis.[34] The choice experiment was pilot tested on ten HCWs prior to final administration in order to evaluate comprehension and respondent burden. Once piloted, the questionnaire was administered to a convenience sample of hospital employees at two large public hospitals. Subjects were recruited through departmental meetings and on hospital wards. Surveys were distributed by a local research assistant and collected within three days of distribution.

The survey collected basic demographic information, provided context and instructions for the choice tasks, and contained 12 BWS choice sets. Each of the 12 choice sets presented the respondent with five attribute levels (see Table 2 for a sample choice set). From each set of five attribute levels, respondents chose the best and the worst attribute level based on their own personal preference for participation in an active TB case finding program. This process was then repeated with a subsequent choice set containing a different set of attribute levels.

**Table 2:** A sample best worst scaling choice set.

Best (most preferred)		Worst (least preferred)
	Location: in another community	
	Test done by a registered nurse	
☑	Wait time: none	
	Test done by an allied health worker	
	Cost: R300	☑

Participants were asked to select the best (most preferred) feature and the worst (least preferred) feature of an active tuberculosis case finding program from the options provided.

### Statistical analysis

BWS data were coded using Latent Gold Choice Version 4.5.0 (Statistical Innovations. Belmont, MA, USA) for latent class analysis (LCA). A random-effects conditional logit model allowed the estimation of coefficients (or relative preference) for 15 attribute levels relative to an investigator-selected reference level.[25,29] The relative preference of each attribute level is then the mean utility of that attribute level relative to a common reference level. In other words, the relative preference of each attribute level can be seen as a measure of strength and direction of preference for a particular attribute relative to a common reference level.

LCA aims to reveal the number of clusters (or classes) from the study sample that best predict preference heterogeneity among respondents.[35] After initial analysis with a one-class model, models were tested with one- to six-classes, and assessed for model fit using the log likelihood function, Akaike information criterion (AIC) and Bayesian information criterion (BIC). The optimal number of classes was determined when an additional class would not significantly improve the model fit. Individual characteristics (covariates) of HCWs, such as age, sex, and occupation, were added to the LCA model to predict class membership.

## Results

### Sample characteristics

One hundred and twenty-five HCWs completed the questionnaire (response rate = 82%). As shown in Table 3, the median age of the respondents was 43.9 (IQR 34.6–52.5) and the respondents were most commonly female (67%) and black (74%). The majority of respondents were nurses (52%), followed by hospital administrators (17%), and physicians (15%). There was a near equal representation between two hospitals (49% from Hospital 1 and 47% from Hospital 2). Two other hospitals were listed as the primary place of employment for the remaining 4% of respondents. Half of the respondents were aware of their hospital’s active TB screening policy and 17% had been tested for active TB in the past year. (Summaries of all respondent characteristics are available in S1 Table. The questionnaire response data are available in S2 Table)

**Table 3:** Characteristics of the respondents (n = 125).

Characteristic		N	%
**Gender**			
	Female	84	67
**Age**			
	**Median** = 43.9 (**IQR**: 34.6–52.5)		
**Race**			
	Black	92	74
	White	28	22
	Coloured	4	3
	South Asian	1	1
**Does someone in your household currently have active TB?**			
	Yes	4	3
**Hospital of employment**			
	Hospital 1	61	49
	Hospital 2	59	47
	Other	5	4
**Occupation**	Nurse	65	52
	Administrator	21	17
	Physician	19	15
	Cleaning staff	6	5
	Aid/Porter	4	3
	Allied health worker	3	2
	Security officer	3	2
	Medical student	2	2
	Driver	2	2
**When were you last assessed for active TB?**			
	Never	54	43
	More than one year ago	41	33
	Less than one year ago	21	17
	Don’t remember	9	7
**Are you aware of your hospital’s current TB policy?**			
	Yes	62	50

### Model estimation

The reference level in this study was set as “wait time equal to six hours”, the least preferred attribute level in the initial analysis. In relation to this level, respondents’ strongest preference was for an active case finding program at no cost (mean: 5.71), followed by assurance of confidentiality (5.12) and no wait time for the consultation (5.08). There was a strong preference against paying a fee of R300 (0.32) and the absence of confidentiality (0.33) in the testing process. Respondents preferred to be tested at the occupational health unit at one’s workplace (4.58) but were indifferent to being tested by a physician (4.30) or a nurse (4.30). However, respondents preferred to be tested by a physician or nurse rather than an allied health worker (2.89)(Table 4). All model estimates were statistically significant at an alpha of 0.05.

**Table 4:** Relative preferences for attribute levels.

Attribute	Attribute Level	Relative Preference, mean (SE)	p value
Test location	At work	4.58 (0.16)	<0.0001
	Own community	3.09 (0.16)	<0.0001
	Another community	2.07 (0.15)	<0.0001
Wait time (hours)	0	5.08 (0.16)	<0.0001
	2	2.68 (0.16)	<0.0001
	4	1.67 (0.14)	<0.0001
Cost (Rand)	0	5.71 (0.17)	<0.0001
	100	2.29 (0.15)	<0.0001
	200	1.44 (0.14)	<0.0001
	300	0.32 (0.14)	0.018
Tested by	Physician	4.30 (0.16)	<0.0001
	Nurse	4.30 (0.16)	<0.0001
	Allied health worker	2.89 (0.16)	<0.0001
Confidentiality	Yes	5.12 (0.17)	<0.0001
	No	0.33 (0.13)	0.011

**Note:** Wait time = 6 hours, used as the relative reference level; random-effects conditional logit model, alpha <0.05

### Latent class analysis

**Fig 1:**
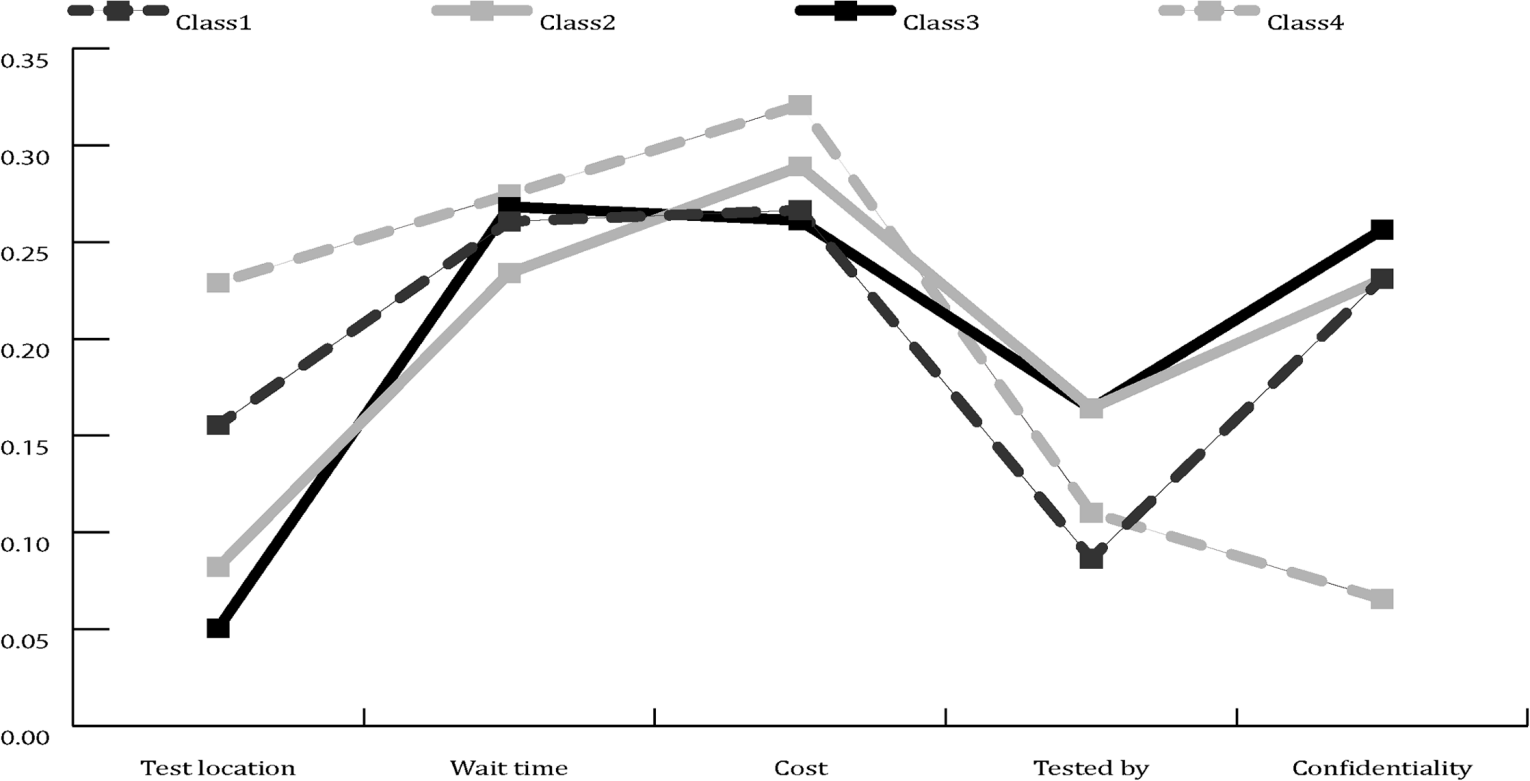
Relative importance of attributes. Rescaled to sum to 1 within each class.

Based on model fit statistics, four classes emerged from this model, and covariates were used to predict class membership **(** Table 5 **).** Relative preferences were then calculated for each attribute level in each class **(** Table 6 **)**. Gender (p < 0.01), occupation (p = 0.01) and the time since a previous TB test (p < 0.01) were statistically significant predictors of class membership. The relative importance of each attribute was determined for the four classes (Fig 1).

**Table 5:** Latent class analysis: Four-class model of variable coefficients and probability of class membership.

	Class1		Class2		Class3		Class4		p value
Intercept	2.05		0.89		-2.39		-0.55		0·0096
Class Size (proportion of sample)	0.55		0.20		0.15		0.10		
	Coefficient	Probability^1^	Coefficient	Probability^1^	Coefficient	Probability^1^	Coefficient	Probability^1^	p value
**Age**									
< = 40 year	-0.15	0.39	-0.044	0.32	-0.34	0.49	0.54	0.42	0.49
>40 years	0.15	0.61	0.044	0.68	0.34	0.51	-0.54	0.58	
**Sex**									
Female	-0.080	0.68	1.55	0.91	-0.76	0.57	-0.71	0.25	0.0027
Male	0.080	0.32	-1.55	0.09	0.76	0.43	0.71	0.75	
**Hospital of employment**									
Hospital 2	-0.56	0.41	0.14	0.59	-0.11	0.56	0.54	0.42	0.098
Hospital 1 & Others	0.56	0.59	-0.14	0.41	0.11	0.44	-0.54	0.58	
**Occupation**									
Administrators & Others	0.47	0.34	0.30	0.40	1.83	0.44	-2.60	0.00	0.012
Nurse	0.22	0.56	-2.08	0.48	2.10	0.56	-0.24	0.25	
Physician	-0.69	0.10	1.78	0.12	-3.92	0.00	2.84	0.75	
**Last assessed for TB**									
< 1year	0.14	0.13	2.61	0.44	-2.81	0.00	0.056	0.08	0.0071
> 1 year	0.30	0.42	-1.92	0.08	0.92	0.12	0.70	0.66	
Never	-0.44	0.45	-0.69	0.48	1.89	0.88	-0.75	0.26	
**Awareness of employer TB policy**									
Yes	-0.12	0.52	0.44	0.61	-0.73	0.24	0.40	0.49	0.11
No	0.12	0.48	-0.44	0.39	0.28	0.76	-0.40	0.51	

^**1**^Probability is interpreted as the probability that class membership is predicted by the given covariate. P-values represent the statistical significance of the covariate as a predictor of class membership.

**Table 6:** Latent class analysis: Relative preferences for attribute levels in a four-class model.

		Relative Preferences, mean (SE)			
Attribute	Attribute Level	Class 1	Class 2	Class 3	Class 4
Test location	At Work	7.77 (0.36)	3.31 (0.40)	2.28 (0.40)	3.97 (0.49)
	Own community	4.13 (0.32)	3.07 (0.39)	1.80 (0.39)	3.90 (0.51)
	Another community	2.86 (0.24)	1.80 (0.34)	1.71 (0.36)	0.34 (0.43)
Wait time	0	8.23 (0.36)	4.31 (0.41)	3.02 (0.39)	4.36 (0.51)
(hours)	2	3.56 (0.29)	1.91 (0.37)	2.17 (0.38)	3.34 (0.51)
	4	1.60 (0.21)	0.86 (0.34)	0.97 (0.34)	1.31 (0.45)
	0	9.13 (0.38)	4.88 (0.40)	3.59 (0.40)	4.69 (0.51)
Cost	100	3.28 (0.26)	1.61 (0.36)	1.44 (0.35)	1.97 (0.45)
(Rand)	200	1.91 (0.22)	1.17 (0.35)	0.94 (0.35)	0.22 (0.41)
	300	0.71 (0.22)	-0.26 (0.32)	0.65 (0.34)	-0.41 (0.43)
Tested by	Physician	6.54 (0.36)	4.44 (0.40)	3.91 (0.39)	1.83 (0.49)
	Nurse	6.49 (0.33)	3.03 (0.39)	4.46 (0.42)	2.87 (0.49)
	Allied health worker	3.82 (0.28)	1.42 (0.36)	2.61 (0.39)	3.58 (0.50)
Confidentiality	Yes	8.27 (0.36)	4.44 (0.42)	4.18 (0.40)	2.07 (0.48)
	No	0.98 (0.22)	0.19 (0.33)	1.29 (0.36)	1.03 (0.46)

Class one members, representing 55% of the sample, were predicted to comprise of female nurses, working at Hospital 1. Cost, wait time and confidentiality were the attributes of greatest relative importance to this class and the occupation of the HCW providing the TB testing was of least relative importance to this class compared to the other classes. Class one preferred active TB case finding in their OHU (7.77) and were indifferent to whether the testing was done by a physician (6.54) or nurse (6.49).

Class two members, representing 20% of the sample, were characterised as female nurses and administrators who were largely aware of the TB policy at their hospital of employment. Cost, wait time and the level of confidentiality were also of greatest relative importance to this class. Class two was essentially indifferent to whether their testing was provided at the OHU (3.31) or a clinic in their community (3.07) and had a strong preference for no cost (4.88), no wait time (4.31) and assurance of confidentiality (4.44).

Class three members, representing 15% of the sample, were predicted to be nurses and administrators, who had never been previously tested for TB, and were not aware of their hospital TB policy. Confidentiality had the strongest relative preference and the location of testing had the lowest relative preference for class three members compared to the other classes. Following the assurance of confidentiality (4.18), the class had a strong preference for testing at no cost (3.59).

Class four membership, representing 10% of the sample, was predominately comprised of male physicians who had been previously tested for TB but more than a year ago. Members of class four had the greatest relative preference for the location of testing, wait time and cost while the lowest relative preference for confidentiality. Class four members also preferred testing at no cost (4.69) and with no wait (4.36) but were indifferent as to whether the testing was conducted at their OHU (3.97) or at a clinic in their community (3.90).

## Discussion

This is the first published study to quantify the preferences of HCWs for active TB case finding programs. In addition to supporting international recommendations that testing HCWs for TB should be offered at no cost by hospital occupational health services,[17] these results illustrate that participation in case finding programs by this high-risk population could be improved by offering the services in a manner that eliminates long waits and that protects confidentiality for HCWs. Recent WHO/ILO/UNAIDS guidelines aimed to improve HIV and TB testing, treatment and support for HCWs noted that the evidence in their systematic review was still weak in supporting the workplace as being the preferred choice for such programs.[21] Some authorities expressed concern that HCWs might prefer to go offsite for such care.[14] As TB is an occupational disease, with potentially devastating consequences for patients and co-workers as well as the HCW’s family, the fact that the OHU was indeed a preferred location by all HCWs, is an important finding from the perspective of TB infection control and occupational health. In light of the recent guideline recommendations to develop and implement programmes for regular, free, voluntary, and confidential testing for TB for HCWs,[21] the results of this study thus help support effective allocation of resources to OHUs to test HCWs for TB, improving the health of HCWs and the populations they serve.

In a recent study in South Africa and a qualitative paper on HCW attitudes toward TB testing in Uganda, [36,37] HCWs similarly expressed concerns that a TB diagnosis would not be maintained confidential by supervisors and colleagues would infer a positive HIV status. Congruent with the findings in our study, the perception of confidentiality during TB case finding, particularly in environments with a high HIV prevalence, continues to impede the utilization of TB services. As identified by our LCA, class three contains the greatest proportion of HCWs who have not previously been tested for TB. This same class had the highest relative preference for testing confidentiality. By quantifying this heterogeneity in HCWs’ preferences, the findings of this study enable case finding programs to effectively align resources to optimize service utilization. It is imperative to not only protect HCW’s confidentiality during case finding but to ensure that these attributes are well communicated within the workforce, particularly those segments of the workforce who have expressed the most concern.[38]

Questions regarding HIV status were not included in an effort to respect participant confidentiality. Although HIV stigma in the workplace exists in the healthcare setting,[39] international policies exist that aim to offer additional protection to these workers. These policies are often unofficial at the facility level. For example, an occupational health nurse at one study hospital stated that she always recommends voluntary relocation of HIV positive HCWs to lower-risk workplaces to prevent TB exposure.

There is a strong relationship between the provision of health-promoting facilities at the workplace and the health-promoting activity of employees.[40] Designing OHU services that are attuned to the preferences of the workforce should increase the utilization of these services.[41] Early clinical detection of active TB reduces the risk of poor outcomes, negative health sequelae and adverse social and economic consequences of TB.[42] Early detection also reduces TB transmission by shortening the duration of possible exposure before treatment.[42]

The application of a BWS choice experiment in this context provides valuable and unique insight into the health-seeking behaviours of HCWs. However, the results of this study must be interpreted in the context of the study design. Similar to other choice experiments, the results of this questionnaire evaluated stated preferences and did not evaluate the choices that HCWs actually make. However, we assume that the findings presented here do reflect what their actual choices have been or would be. The survey was administered in the two largest hospitals in the province. The characteristics of our study sample do not differ from the HCW population in the Free State.[43] The attributes used are not inclusive of all possible attributes. We recognize there may be alternative attributes that also influence decision-making, although early qualitative work aimed to minimize this risk.

Recent literature has highlighted increasing merit of effective large scale case finding programs in populations at high-risk of TB.[42,44] Our findings address many of the potential implementation challenges by eliciting decision-making preferences of the target population.[44,45] Given the tremendous economic burden and social costs of TB, there are considerable potential economic, social, and public health benefits to be gained from improving the control and prevention of TB through more frequent and timely case finding. Specifically, the findings presented here shed light on preferences of HCWs, enabling policy makers to optimally design TB programs for HCWs in this region. As indicated by our results, TB testing for HCWs in South Africa should be free, highly confidential, with minimal wait times and available at the workplace. Attention to heterogeneity in preferences should optimize TB testing utilization of target HCW populations.

## Supporting Information

S1 Table Full summary of respondent characteristics. (DOCX)

S2 Table Questionnaire response dataset. (XLSX)
