# Supplementary material for: Healthcare Worker Preferences for Active Tuberculosis Case Finding Programs in South Africa: A Best-Worst Scaling Choice Experiment
Source: PLoS One. 2015 Jul 21;10(7):e0133304. doi: 10.1371/journal.pone.0133304 (PMC4511419; doi:10.1371/journal.pone.0133304)
Supplement: S1 Table — (DOCX) [file pone.0133304.s001.docx]

**Table S1** Full summary of respondent characteristics

| **Variable** | | **N** | **%** |
| --- | --- | --- | --- |
| **Version of questionnaire** | |  |  |
|  | 1 | 35 | 23 |
|  | 2 | 35 | 23 |
|  | 3 | 40 | 26 |
|  | 4 | 42 | 28 |
|  |  |  |  |
| **Age** | |  |  |
|  | <20 | 1 | 1 |
|  | 21-25 | 6 | 4 |
|  | 26-30 | 18 | 12 |
|  | 31-35 | 16 | 11 |
|  | 36-40 | 20 | 13 |
|  | 41-45 | 20 | 13 |
|  | 46-50 | 20 | 13 |
|  | 51-55 | 23 | 15 |
|  | 56-60 | 19 | 13 |
|  | 60+ | 9 | 6 |
|  |  |  |  |
| **Sex** | |  |  |
|  | Female | 104 | 68 |
|  | Male | 48 | 32 |
|  |  |  |  |
| **Race** | |  |  |
|  | Black | 110 | 72 |
|  | White | 35 | 23 |
|  | Coloured | 6 | 4 |
|  | South Asian | 1 | 1 |
|  |  |  |  |
| **Active TB** | |  |  |
|  | No | 148 | 97 |
|  | Yes | 4 | 3 |
|  |  |  |  |
| **Workplace** | |  |  |
|  | Hospital 1 | 83 | 55 |
|  | Hospital 2 | 64 | 42 |
|  | Hospital 3 | 3 | 2 |
|  | Hospital 4 | 1 | 1 |
|  | Hospital 5 | 1 | 1 |
|  |  |  |  |
| **Occupation** | |  |  |
|  | Nurse | 74 | 49 |
|  | Administrator | 32 | 21 |
|  | Physician | 23 | 15 |
|  | Household Aid | 9 | 6 |
|  | Cleaning staff | 7 | 5 |
|  | Driver | 3 | 2 |
|  | Allied health professional | 3 | 2 |
|  | Missing | 1 | 1 |
|  |  |  |  |
| **Last assessed for TB** | |  |  |
|  | Never | 65 | 43 |
|  | More than 1 year | 54 | 36 |
|  | Less than 1 year | 24 | 16 |
|  | Don’t know | 8 | 5 |
|  | Missing | 1 | 1 |
|  |  |  |  |
| **Aware of TB policy** | |  |  |
|  | Yes | 77 | 51 |
|  | No | 75 | 49 |
